# Correction: Specific transfection of inflamed brain by macrophages: A new therapeutic strategy for neurodegenerative diseases

**DOI:** 10.1371/journal.pone.0306688

**Published:** 2024-07-02

**Authors:** The PLOS ONE Editors

The corresponding author requested correction of panel B of Fig 3. Please see the correct Fig 3 and its caption here.

**Fig 3:**
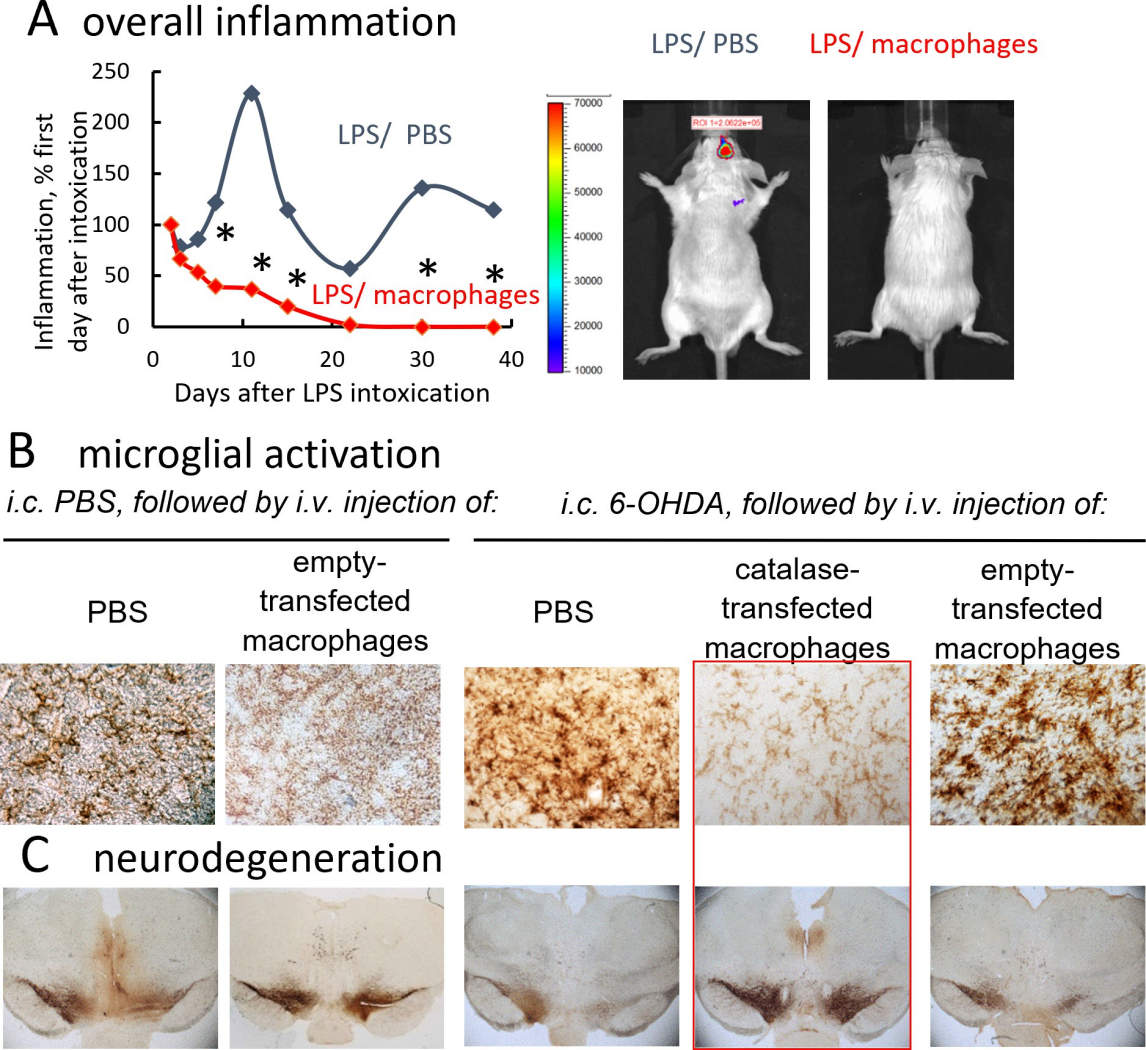
Anti-inflammatory and neuroprotective effects of catalase-transfected macrophages in PD murine models. (A): LPS-induced encephalitis in BALB/C mice were injected *i*.*v*. with catalase-transfected macrophages (red curve), or PBS (blue curve). IVIS images over 40 days were taken ten minutes after intraperitoneal (*i*.*p*.) injection of a XenoLight RediJect probe for inflammation. The chemiluminescent signal was quantified and presented as radiance ratios of treated animal after 24 hours after LPS injection and at various times thereafter. Genetically modified macrophages caused prolonged decreases of neuroinflammation in LPS-intoxicated mice. IVIS representative images at day 30 are shown. (B) and (C): BALB/c mice were *i*.*c*. injected with 6-OHDA. Forty-eight hours later animals were *i*.*v*. injected with catalase-transfected macrophages, and 21 days later they were sacrificed, and mid-brain slides were stained for expression of B: CD11b, a marker for activated microglia or C: TH, a marker for dopaminergic neurons. Whereas 6-OHDA treatment caused significant microglia activation and neuronal loss, administration of catalase-transfected macrophages dramatically decreased oxidative stress and increased neuronal survival. Administration of empty vector transfected macrophages did not affect microglia activation, or number of dopaminergic neurons in in mice with brain inflammation. Statistical significance (shown by asterisk: *p* < 0.05) was assessed by a standard t-test compared to mice with *i*.*c*. LPS injections followed by *i*.*v*. PBS injections (healthy controls). Values are means ± SEM (N = 4).

## References

[1] Haney MJ, Zhao Y, Harrison EB, Mahajan V, Ahmed S, He Z, et al. (2013) Specific Transfection of Inflamed Brain by Macrophages: A New Therapeutic Strategy for Neurodegenerative Diseases. PLoS ONE 8(4): e61852. 10.1371/journal.pone.0061852 23620794 PMC3631190

